# The Many Applications of Engineered Bacteriophages—An Overview

**DOI:** 10.3390/ph14070634

**Published:** 2021-06-30

**Authors:** Bryan Gibb, Paul Hyman, Christine L. Schneider

**Affiliations:** 1 Department of Biological and Chemical Sciences, Theobald Science Center, Room 423, New York Institute of Technology, Old Westbury, NY 11568-8000, USA; 2 Department of Biology and Toxicology, Ashland University, 401 College Ave., Ashland, OH 44805, USA; phyman@ashland.edu; 3 Department of Life Sciences, Carroll University, 100 North East Ave., Waukesha, WI 53186, USA

**Keywords:** bacteriophage, phage therapy, engineered phage, imaging agent, pathogen detection

## Abstract

Since their independent discovery by Frederick Twort in 1915 and Felix d’Herelle in 1917, bacteriophages have captured the attention of scientists for more than a century. They are the most abundant organisms on the planet, often outnumbering their bacterial hosts by tenfold in a given environment, and they constitute a vast reservoir of unexplored genetic information. The increased prevalence of antibiotic resistant pathogens has renewed interest in the use of naturally obtained phages to combat bacterial infections, aka phage therapy. The development of tools to modify phages, genetically or chemically, combined with their structural flexibility, cargo capacity, ease of propagation, and overall safety in humans has opened the door to a myriad of applications. This review article will introduce readers to many of the varied and ingenious ways in which researchers are modifying phages to move them well beyond their innate ability to target and kill bacteria.

## 1. Introduction

Bacteriophages or phages have been studied and used for just over 100 years [1,2]. During most of that time, in at least some parts of the world, phages have been used as antibacterial agents. This application is commonly called phage therapy. With the increasing levels of antibiotic resistant bacteria seen world-wide, interest in phage therapy is also increasing, especially in parts of the world that had abandoned it in favor of antibiotics [3,4].

Phage therapy has mostly used phages as they are found in nature since at least some phages for any particular host species are obligately lytic meaning they lack the ability to perform lysogeny. There are, however, some bacterial hosts such as *Clostridioides difficile* (formerly *Clostridium difficile*) for which only temperate phages have been isolated [5]. Additionally, the natural presence of bacteriophage resistant strains or subtypes [6] had led some researchers to explore ways to improve the killing efficiency of phages through genetic engineering (Section 2). Genetically engineered phages have been used to improve existing methods to kill bacterial hosts, provide phages with entirely novel mechanisms to kill host cells, and alter gene expression of targeted bacterial hosts (Section 3).

In addition to using phages to kill bacteria, researchers have creatively modified phages for applications completely unrelated to the normal phage life cycle. For example, vaccines can be designed using phages that either display a protein antigen from a human pathogen or a cancer cell on their surface or carry the genetic information to encode the antigen (Section 4.4). Phages screened by a process called phage display (Section 4) can identify novel peptides and proteins that bind to a target material such as a cancer cell. These peptides can then be displayed on the surface of a phage directing the phage to deliver a chemotherapy drug to the tumor cell (Section 4.2). Additionally, phages can be used to detect specific bacteria even in a mixture of many bacterial species (Section 5).

While some of these applications can be done with other materials such as antibodies, phages have several properties that make them especially useful in developing new therapeutic agents.

**Figure 1:**
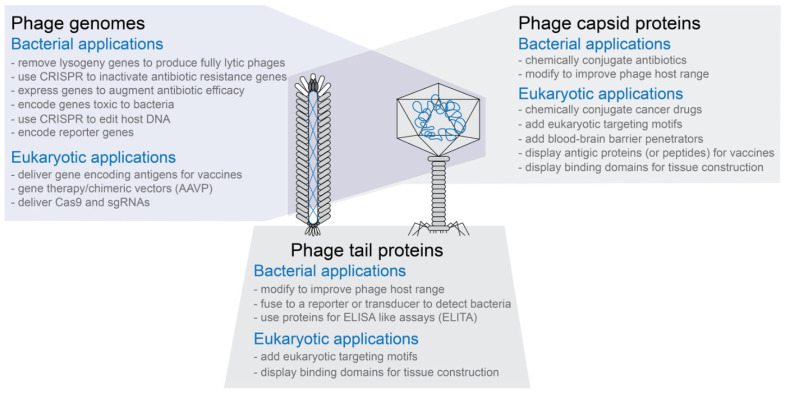
Overview of engineered phages. Both filamentous and tailed phages have been modified to create phages with novel functions. The three modification types are not exclusive of each other. That is, for example, phages in which both the capsid and tail fibers have been modified have been designed and produced.

- Phages are typically very specific in the types of bacterial cells they target
- Phages are relatively easy and inexpensive to propagate on bacterial hosts using well established protocols
- Phage capsids are highly stable, often resistant to changes in pH and temperature
- Phage capsids have multiple proteins that can be targeted for modification without necessarily inactivating the phage capsid’s functions (see Figure 1)
- Phage capsids protect the DNA or RNA packaged in them
- Phage capsids or modified phage capsids can be used to package non-phage nucleic acid, protein, or other types of materials (see Figure 1)
- Phage genomes are small compared to bacterial or eukaryotic genomes and are often relatively easy to modify using genetic engineering techniques
- In vitro packaging systems have been created to move genetically modified genomes into capsids as well as transformation protocols to move genomes directly into cells for expression
- Phage display technology can be used to create phages with novel binding properties even to eukaryotic targets
- For human treatment applications, phages are generally recognized as safe (GRAS) agents

Given these advantages, it is not surprising that phages are being used as therapeutic agents for a wide variety of both bacterial and eukaryotic related applications. In this overview article to this special issue of *Pharmaceuticals*, we briefly describe many of these applications. More details can be found in the other articles within this issue.

## 2. Methods to Genetically Engineer Phages

Although the sheer abundance of phage in the environment allows for the use of naturally occurring bacterial viruses in many therapeutic approaches, phages can be enhanced using genetic engineering approaches (for review see Pires [7]). These techniques allow researchers to increase phage killing efficacy or to introduce other desirable properties, such as expanded host range, increased biofilm degradation, elimination of lysogeny, and the addition of genes to arm phages with secondary antimicrobial payloads [8,9,10,11,12]. And as we will describe below, these techniques and others can be used to alter phages in ways that extend their ability to infect bacteria or add other new functionality for therapeutic use.

**Figure 2:**
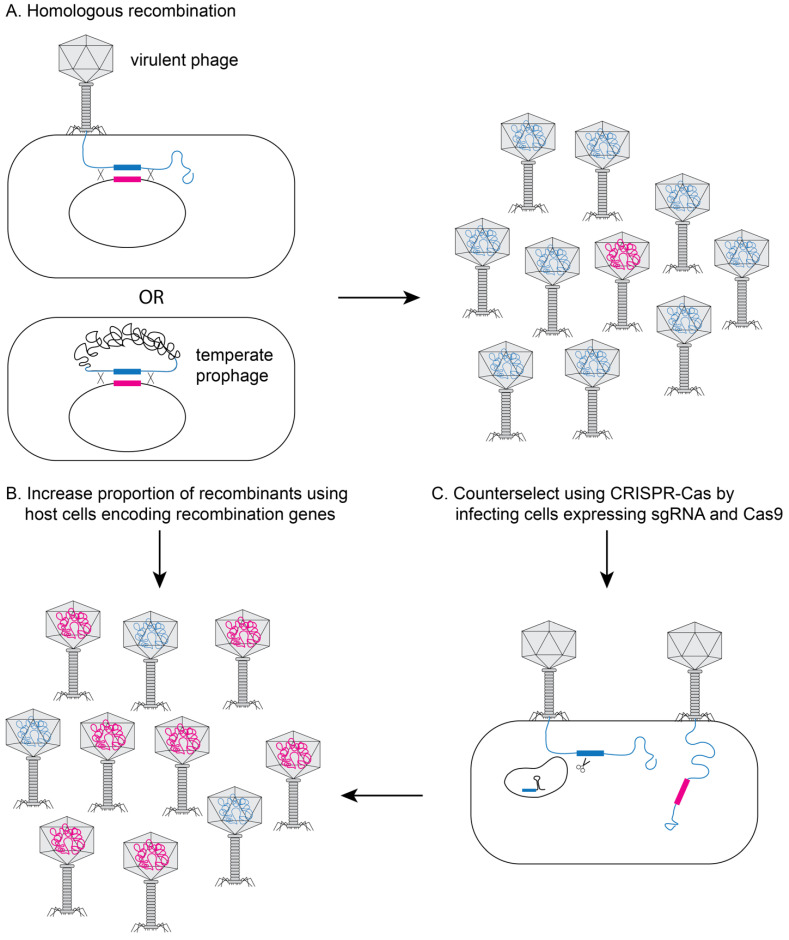
Genetically engineering phage in bacteria. (**A**) During infection with a virulent phage or in a lysogen for a temperate phage, the phage DNA can recombine with plasmid DNA containing regions of homology flanking the edited section of DNA (pink section). Typical recombination rates are low (10^−10^–10^−4^), yielding very few recombinant phage (pink DNA in head). The proportion of recombinant phage can be increased by using host bacterial cells that express recombination genes (e.g., RecE/RecT or λ-red proteins) (**B**) and/or by using CRISPR/Cas9 to inactivate non-recombinant DNA (**C**).

One of the most widely used approaches for phage engineering is homologous recombination, where a heterologous segment of DNA is recombined with the phage genome at sites of homology within a bacterial host (see Figure 2A) [13,14,15]. The efficiency of recombination is very low (10^−10^–10^−4^) [13,14,15], so an efficient screening method is necessary to identify recombinant phages [16,17,18]. The recombination efficiency is vastly improved if the phages to be edited are transformed into bacterial strains that express high levels of heterologous recombination proteins such as RecE/RecT-like proteins [18] or the lambda red system (see Figure 2B) [19,20]. The efficiency of recovering recombinant phage can be further improved using CRISPR-Cas to counter-select against non-recombinant phage (see Figure 2C) [21,22,23,24,25,26,27,28]. 

**Figure 3:**
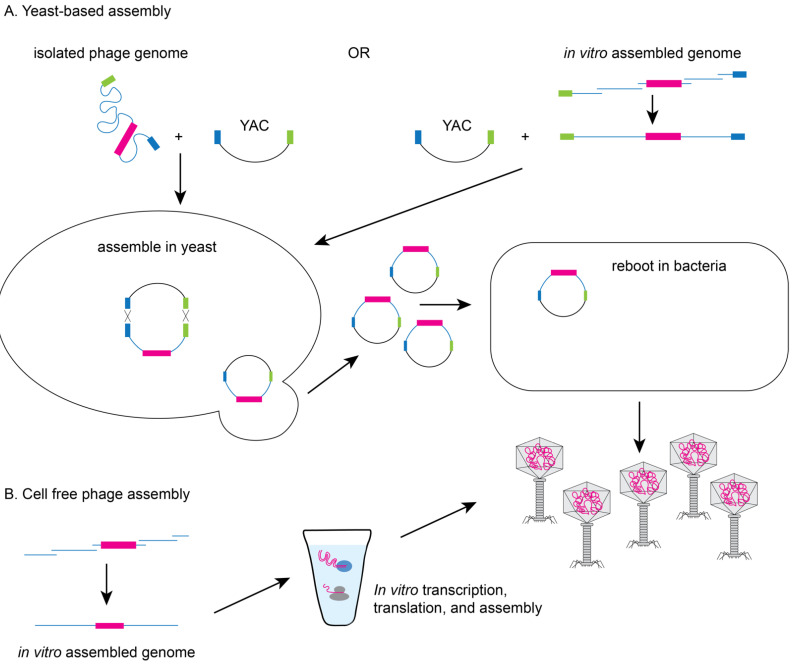
Yeast-based or cell-free phage assembly. (**A**) To avoid issues of toxicity with phage genes, modifications can be made to phage genomes in yeast using TAR based cloning. Phage genomes (isolated from a modified phage or assembled in vitro) are electroporated into yeast with a linearized yeast artificial chromosome (YAC) containing hooks (blue and green boxes) to facilitate recombination. YACs containing the modified phage genomes can be electroporated into bacteria to produce recombinant phage particles. (**B**) Alternatively, phage particles can be produced from assembled recombinant phage genomes using cell-free transcription/translation systems.

Yeast-based or in vitro phage genome assembly methods have been developed to avoid the possible toxic effect of phage replication on the bacterial host. Since yeast cells have an efficient recombination system [9] and phage genes are not toxic to yeast, *Saccharomyces cerevisiae* can facilitate the assembly of synthetic genomes by transformation associated recombination (TAR) cloning and the assembled genomes can then be electroporated into a permissive bacterial host for rebooting (see Figure 3A) [11,29]. Alternatively, phage genomes can be assembled in vitro from synthesized DNA fragments that have been assembled by recombination or methods such as Gibson assembly prior to transformation into a permissive bacterial host [9]. Finally, cell-free transcription-translation systems can create the phage or virus-like particles from DNA in vitro overcoming the need for a highly competent bacterial host (see Figure 3B). The Noireaux laboratory has developed cell-free systems to successfully replicate, synthesize, and assemble infectious T7, phiX174, MS2, and even T4 phage which has a 170 kb genome [30,31,32,33].

## 3. Genetically Engineered Phages for Anti-Bacterial Applications

### 3.1. Enhancing Phage Therapy

The use of genetically modified or synthetic phages and phage derived products can extend the therapeutic potential of phage therapy. Work out of the Stevens laboratory shows the benefits of phage engineering to enhance the therapeutic potential of a bacteriophage [8,10,34]. Phage φEf11 was first isolated by prophage induction from a clinical isolate of *Enterococcus faecalis* in an oral biofilm [34]. Zhang and colleagues removed genes associated with lysogeny to make the phage fully lytic and insensitive to repression, transferred genes associated with DNA replication and packaging from another related phage to enhance lytic growth and extend the host range [10], and demonstrated its efficacy in killing vancomycin-resistant *E. faecalis* in a biofilm [8]. More recently, the first clinical application of engineered bacteriophages was used to treat a cystic fibrosis patient with a disseminated *Mycobacterium abscessus* infection [35]. Despite having a bank of over 1800 mycobacterial phages, only one effectively killed the clinical isolate of *M. abscessus* (GD01). Two other phages were found to infect the strain, but poorly. Using bacteriophage recombineering of electroporated DNA (BRED), the repressor gene was removed from one of the phages, which greatly improved the lytic activity of the phage. Host range mutants of the third phage were isolated that increased the ability to infect GD01. The cocktail of the three phages was found to efficiently kill GD01 in vitro. Nine days after initiation of phage therapy, the patient was able to leave the hospital, and after 7 months of treatment (IV and topical phage therapy and antibiotics), there was significant improvement in the lungs, liver, and skin.

### 3.2. Modifying the Gut Microbiome

As research expands our understanding of microbial communities in the gut and the impact of these microbes on human health, researchers have continued to seek ways to modulate the composition of the microbes that reside in the gut. The use of virulent bacteriophages to alter the microbial community of the gut has been attempted in animal models. Hsu and colleagues tested the effect of phages on germ-free mice colonized with a ten-member model microbiota comprised of commensal bacterial species known to colonize the human gut. They targeted each member individually with phages, which reduced but did not fully eliminate the target bacteria from the population [36]. Bacterial members of the community are held in a balance by a series of inter-species signals that positively or negatively influence the growth of other members. The authors observed that the depletion of a target species by phage predation altered the population of non-target species due to the corresponding decrease in the positive and negative signals contributed by the target species. They also saw resistance to phages emerge over the course of their experiment. Within two days, 28% of the *E. faecalis* population were phage resistant and by ten days 68% were resistant [36]. This work demonstrates the ecological importance of bacteriophages in modulating the gut microbiome, but the development of resistance is a lingering concern for the potential use of virulent phages to therapeutically alter the microbiome. Although this work demonstrates that we still have much to learn about the microbial community of the gut and its effects on human health, several companies are now pursuing the use of genetically modified phages to alter the composition of the microbiome and using phage-based vectors based on CRISPR-Cas systems to alter specific members of a community [37,38].

### 3.3. Altering Phage Host Range

Phages typically infect a limited range of bacterial species and a varying number of strains within each species [39]. This narrow specificity is an advantage in the sense that phages will not disrupt commensal bacteria of the host but demands that each phage be screened against each bacterial target to determine susceptibility prior to treatment. To circumvent this limitation, multiple phages can be mixed into a cocktail that will have a broader range of activity. Alternatively, phages can be genetically altered to expand their host range. The tail fibers of many phages mediate binding to the host and are a primary determinant of host range. Several studies have demonstrated that swapping genomic segments of the tail fiber from two different phages swaps the host range [14,15,40]. Others have increased the host range of filamentous phages by fusing a receptor-binding domain to capsid proteins [41,42]. Yehl and colleagues developed a targeted and high-throughput method to broaden host-range using targeted mutagenesis of well-defined regions of the tail fiber that mediate host recognition. This approach generates a vast amount of diversity. The mutant phage library with expanded host range reduced the emergence of phage-resistant bacterial mutants relative to an infection with the wild-type phage. By screening a library of mutant phages against phage-resistant mutant bacterial strains, phages capable of infection were enriched and the specific mutations that permit the expanded host range characterized [43]. 

### 3.4. Altering Antibiotic Sensitivity

Beyond direct phage-induced lysis of bacteria, various phage components can also be engineered to carry payloads that enhance the bactericidal activity of antibiotics (see Figure 1). In one example, Lu and Collins modified the lysogenic phage M13mp18 to overexpress *lexA3*, which represses the SOS DNA repair system. Administration of ofloxacin, a quinolone antibiotic which induces DNA damage, to a strain infected with the *lexA3*-producing phage improved bactericidal activity by 4.5 and 2.7 orders of magnitude compared with bacteria treated with ofloxacin alone and ofloxacin with unmodified phage respectively. The modified phage was even effective at killing strains with resistance to the antibiotic [44]. The authors further demonstrated the effectiveness of this method in an in vivo mouse model where mice treated with the antibiotic and modified phage had an 80% survival rate compared with 20% for treatment with ofloxacin alone and 50% with ofloxacin and unmodified phage [44]. In another example, Edgar and colleagues engineered phage λ to carry wild-type versions of the *rpsL* and *gyrA* genes. Bacteria that acquire mutations in these genes are resistant to streptomycin and nalidixic acid respectively. When *Escherichia coli* was lysogenized with the modified phage, the production of the wild-type versions of these genes conferred sensitivity to antibiotics in a dominant manner where the minimum inhibitory concentration (MIC) decreased 8-fold for streptomycin and 2-fold for nalidixic acid [45]. 

Several groups have engineered phages to carry or use CRISPR-Cas systems to disrupt antibiotic resistance genes [46,47]. Citorik and colleagues [47] designed filamentous phage-based phagemid constructs targeting β-lactamase genes, which confer resistance to β-lactam antibiotics. Once introduced into cells, RNA-guided nucleases bind to specific genetic sequences within the β-lactamase gene and generate a double-strand break, which leads to cell death or loss of the plasmid containing the β-lactamase gene. When *E. coli* were treated with phage particles encoding a β-lactamase targeting system, viable cell counts of cells containing the gene decreased by 2 to 3 orders of magnitude while cells lacking the β-lactamase gene were unaffected. The authors further demonstrated the specificity of this approach by targeting a quinolone-resistant strain of *E. coli,* where resistance was conferred by a single-nucleotide mutation in the chromosomal *gyrA* gene encoding DNA gyrase. Phage particles encoding a *gyrA* targeting system were cytotoxic to the resistant *E. coli* strain, but not for the isogenic parental strain. Furthermore, administration of phages targeting a major virulence gene (*eae*) of enterohemorrhagic *E. coli* O157:H7 (EHEC) significantly improved survival relative to controls in a *Galleria mellonella* in vivo infection model [47]. Bikard and colleagues used a similar approach to design a Cas9-based phagemid based on staphylococcal ΦNM1 phage that targets the *aph-3* kanamycin resistance gene in *Staphylococcus aureus* [46]. Infection by this phagemid led to a 4-fold reduction in colony forming units in the kanamycin-resistant bacteria but did not elicit any effect on the kanamycin-sensitive bacteria. They then demonstrated the effectiveness of this method in an infection model against clinical isolates of *S. aureus*, where guide-RNA encoding phagemids selectively killed strains expressing the target genes *mecA* or *sek.*

### 3.5. Delivering Antimicrobials

The exquisite ability of phages to target specific bacteria opens the door to more targeted delivery of antimicrobials. Yacoby and colleagues used filamentous phages as targeted drug carriers to eradicate pathogenic bacteria [48,49]. The authors targeted phage to specific bacteria both by genetically modifying the p8 coat protein of the phage to display a bacterial-specific peptide and by linking antibodies to the phage via an IgG-binding ZZ domain displayed on the minor coat protein of the phage. An inactive form of chloramphenicol, a bacteriostatic antibiotic, was chemically conjugated to the phage via a labile linker. The modified phage carrying chloramphenicol were able to bind the target bacteria while esterases in serum cleaved the linker releasing an active form of chloramphenicol near the target bacteria. This approach increases the local concentration of the drug at the target site, thereby increasing the potency and decreasing the general toxicity as the drug is inactive while conjugated to the phage [49]. Using an improved linker, the authors loaded over 40,000 chloramphenicol molecules/phage, which when tested in vitro completely inhibited the growth of *S. aureus, Streptococcus pyogenes,* and *E. coli* and was nontoxic when injected into mice [48,50]. 

Phages can also be engineered to carry genes known to inhibit bacterial growth and survival. One example is an engineered M13 phage modified to encode toxin genes (*gef* and *chpBK*) that induce cell death during nutritional deficiency. Bacteria lysogenized with the modified M13 phage, which resulted in the expression of either Gef or ChpBK, showed a reduction in colony forming units by 948- and 1579- fold respectively [51]. 

### 3.6. Deploying Targeted CRISPR Editing

CRISPR editing offers the potential to inactivate or modify any gene in a bacterial population in addition to antibiotic resistance genes or to deliver a novel gene to a population in a specific manner without disturbing the beneficial microbiota. Selle and colleagues used genetically modified phage ΦCD24-2 encoding a self-targeting CRISPR to redirect the endogenous type I-B CRISPR-Cas3 system in *C. difficile* toward the bacterial chromosome [52]. Upon infection, the phage-delivered CRISPR activates the endogenous Cas3 protein to processively digest the chromosomal DNA of the bacterial host. The authors found that modified phage carrying the self-targeting crRNA was significantly more effective at killing *C. difficile* than the wild-type bacteriophage, both in vitro and in a mouse infection model. 

Rather than using CRISPR for cutting target DNA, researchers can target a nuclease-deactivated Cas9 (dCas9) to a specific gene to alter its expression. As a proof of principle for in vivo alteration of gene expression of microbes in the gut, Hsu and colleagues engineered phage λ to express dCas9 and a guide RNA targeting the gene for red fluorescent protein (λ::dCas9^rfp^) [53]. When the engineered phage was added to a culture of a reporter strain of *E. coli* containing the genomically integrated RFP gene, RFP fluorescence was significantly reduced relative to the control lacking the targeting crRNA. This repression of RFP was maintained during lysogeny. The authors used this system to optimize an encapsulation strategy to allow phage to reach the gut. They found the phage had a similar effect on RFP expressing *E.coli* in the gut of mice. RFP levels of *E. coli* recovered from the gut of mice infected with λ::dCas9^rfp^ was reduced relative to the non-targeting phage λ::dCas9 and no-phage controls [53]. Studies like these demonstrate the potential of using phage to deliver CRISPR based technologies to edit the DNA of bacteria or to alter their gene expression in situ.

### 3.7. Disrupting Biofilms

Many bacteria can grow in multicellular biofilms where the cells are bound by a polysaccharide extracellular matrix. The extracellular matrix facilitates attachment to surfaces such as living tissue, medical devices, food, industrial equipment, or water pipes. Biofilms typically confer tolerance to antimicrobial agents by providing a physical barrier, but also because innermost cells are less metabolically active [54]. Phages that infect bacterial strains containing a capsule made up of these same extracellular matrix components often produce depolymerases, enzymes that degrade polysaccharides. Phages that do not normally produce depolymerases can have these genes added by genetic engineering. Lu and Collins developed a T7 phage which expresses the biofilm-degrading enzyme dispersin B (DspB) intracellularly during phage infection. The modified phage reduced the bacterial biofilm cell counts by more than 99% and was two orders of magnitude more effective at biofilm breakdown than the wild-type phage lacking dispersin B [55]. 

While the use of depolymerases is an effective strategy for improving phage effectiveness against bacterial biofilms, these enzymes are highly specific in the type of polysaccharide they can degrade. Pei and colleagues took a different approach by engineering T7 phage to encode enzymes that interfere with quorum sensing, the cell-cell communication used by bacteria to facilitate biofilm formation and maintenance. Phage T7aiiA was engineered to carry the acyl-homoserine lactonase gene from *Bacillus anthracis,* a quorum-quenching enzyme that inactivates the quorum-sensing molecule acyl-homoserine. T7aiiA reduced the quorum sensing of *Pseudomonas aeruginosa* in a mixed biofilm with *E. coli* resulting in a reduction in biomass by 75% and 66% at 4 and 8 h respectively, whereas the control T7 phage reduced biomass by 24% and 32% [56].

### 3.8. Killing Bacteria with Endolysins 

Rather than using virulent phages to kill bacteria, several groups have focused instead on the cell wall degrading endolysins encoded by phages. Near the end of the replication cycle, phage encoded endolysins (or lysins) degrade the host peptidoglycan, which causes cell lysis, releasing the assembled phage particles. Purified recombinant endolysins are particularly effective at lysing gram-positive bacteria as the peptidoglycan layer is exposed on the outer surface of the cell. Endolysins are composed of modular domains consisting of N-terminal enzymatically active domains (EADs) and C-terminal cell wall-binding domains (CBDs), which scientists have used to engineer endolysins with improved properties. For example, chimeric endolysins have been shown to have an altered host range and enhanced activity in *Listeria* [57], *Streptococcus* [58], and *Gardnerella* [59]. Landlinger and colleagues used a domain shuffling strategy to engineer endolysins with 10-fold higher bactericidal activity than the wild-type enzyme against species of *Gardnerella*, which are a key component of a multispecies biofilm causing bacterial vaginosis. The most active endolysin was able to kill *Gardnerella* bacteria and dissolve the biofilm without disrupting the remaining vaginal microbiome in thirteen of fifteen patient samples [59].

Endolysins have also been engineered with modifications that facilitate transport of the lysin across the outer membrane of gram-negative bacteria into the periplasm where it can access the peptidoglycan to improve their efficacy against these bacteria [60,61]. Briers and colleagues engineered endolysins fused to peptides that destabilized the lipopolysaccharide outer membrane, to create what they called Artilysins [60]. Optimized Artilysins were shown to reduce titers of multidrug resistant *P. aeruginosa* and *Acinetobacter baumannii* in culture by 4 to 5 logs within 30 min. In an alternative approach, Heselpoth and colleagues exploited a pyocin transport pathway to move the endolysin through the outer membrane. *P. aeruginosa* produces S-type pyocins which are colicin-like bacteriocins used for intraspecies competition. The authors fused the functional domain of a pyocin to endolysin, which they called a lysocin, to facilitate transfer to the periplasm. The lysocin PyS2-GN4 is bactericidal above 0.1 µg/mL after 4 h and capable of sterilizing high concentrations of *Pseudomonas* at nanomolar concentrations. Notably, in contrast to Artilysins, the lysocin was still effective in human serum [61].

Finally, cell wall binding domains can be used as cell targeting molecules without the lysing domain. For example, a cell wall binding domain can be bound to a bactericidal agent such as a silver nanoparticle to produce a targeted antibacterial compound [62].

## 4. Genetically Engineered Phages for Eukaryotic Applications

Although phages have evolved to specifically infect their bacterial hosts, scientists have devised many creative ways to expand the medical potential of phages to include eukaryotic cell targets (for example, see Grigonyte et al. [63]). Several features of phages make them ideal candidates for therapeutic cargo delivery (for a review see [64]). As described earlier, these include the stability of phages, small genomes that are often easy to modify, and ability to display novel peptide sequences on their capsids without compromising capsid function (see Figure 1). Phage display is used in many applications to identify binding peptides to target the phages. This technique, in which a library of binding peptides is inserted into a bacteriophage for screening for the best binding peptides to a target, has been extensively reviewed elsewhere [65,66,67]. Briefly, a population of phages, modified to contain potential binding peptides fused to surface protein of the phage capsid, are exposed to a target material such as a protein or a cell, those with matching binding peptides bind, and the others are washed away. The bound phages are released and grown to produce a new population. This is repeated for 3–5 rounds to select for a population of phages with binding peptides that bind tightly to the target material. Phage display has been successful in producing binding peptides for both organic targets such as cell growth factors [68]; dengue virus [69]; and biotin [70] and inorganic targets such as gold [71]; silica [72]: and semiconductor compounds [73].

### 4.1. Targeting Eukaryotic Cells

Bacteriophages can be modified to identify nonbacterial targets including eukaryotic proteins and tissues by altering the tail fibers or capsid proteins to include eukaryotic targeting motifs using phage display. The short binding peptides (7–12 amino acids) typically identified via phage display can also be replaced with much larger single-chain antibodies (scFv) (200+ amino acids consisting of a single protein with both heavy and light chain binding domains) which are most often directed at organic molecules. For example, Medecigo and colleagues [74] identified scFvs binding human beta-amyloid, a protein that may play a role in Alzheimer’s disease while Orner and colleagues [75] similarly developed peptides binding beta-amyloid using phage display. These latter peptides were shown to not only bind to beta-amyloid but to interfere with its aggregation suggesting that these peptides could be used to better understand the aggregation process that occurs in Alzheimer’s disease. Similar to scFvs, nanobodies are single chain antibodies based on camelid or shark single heavy chain antibodies. These contain just the binding fragment of the longer single-chain antibody. Their small size allows binding to more specific epitopes than larger antibodies. These nanobodies have been studied for use as antitumor and antibacterial therapeutics [76] among other uses. Other proposed uses for peptides and binding proteins developed using phage display include targeting elements for liposomes [77], cell fluorescence imaging [78], PET imaging [79] and combined imaging and drug delivery to lung cancer cells [80].

### 4.2. Delivering Drugs to Eukaryotic Cells

The ability of engineered phages to directly deliver drugs to specific cancer cells has the potential to minimize the side effects and off target toxicity of more traditional cancer therapies (for reviews see [81,82]). Phages can be linked to drugs that have low water solubility which allows for enhanced delivery and a lower dose to be administered. Endocytosis of the phage particles then delivers either the genetic or drug cargo specifically into the targeted eukaryotic cell. As proof of principle, Bar and colleagues reported a greater than 1000-fold improved potency of hygromycin carried on phages as compared to free drug treatment in vitro of the human breast adenocarcinoma SKBR3 cells [83]. Furthermore, Du and colleagues coupled phages targeting the human hepatocarcinoma cell line BEL-7402 with doxorubicin and observed a reduction in tumor growth and enhanced long-term survival in xenografted mice treated with drug-loaded phages as compared to free drug treatment [84]. Engineered phages have also been designed to deliver photosensitizers to cancer cells, permitting targeted killing of cancer cells following light activation [85].

The targeting achievable with bacteriophages can also allow drugs to be delivered to body compartments that normally exclude drugs such as the brain. The tight junctions of the cells that comprise the blood-brain barrier prevent 98% of small (<400 Da) and 100% of large molecules (>400 Da) from entering the brain [86]. Phages have successfully been designed to shuttle drug cargos across the blood brain barrier using Trojan horse strategies. For example, by conjugating a cell penetrating peptide from the Tat protein of human immunodeficiency virus type-1 to the exterior of P22 phage particles carrying the snail neuropeptide ziconotide, Anand and colleagues demonstrated transportation of ziconotide in several in vitro blood-brain barrier models [87]. Apawu and colleagues conjugated the synthetic peptide angiopep-2 to the capsid of MS2 containing an MRI detectable Mn^2+^ coordinated porphyrin ring and demonstrated these nanoparticles crossed the blood-brain barrier in rats [88]. These studies and others demonstrate the potential for creating phage-based nanoparticles capable of crossing the blood-brain barrier to deliver a variety of cargos to enable diagnosis and treatment of intractable brain disorders like tinnitus, Parkinson’s, and Alzheimer’s disorders.

### 4.3. Delivering Genes to Eukaryotic Cells

Conventional gene therapy approaches have typically relied on eukaryotic viral vectors, but interest in using phage vectors or vectors that are hybrids of eukaryotic and phage viruses is increasing due to key benefits they offer regarding targeting, cargo capacity, and safety (for reviews see [89,90]). Bacteriophages have a large cargo capacity relative to most mammalian viruses and, as discussed above, many can be easily engineered to express eukaryotic cell targeting motifs that also mediate entry via receptor mediated endocytosis. Bacteriophages might also prove safer than mammalian viral vectors given their lack of natural tropism for eukaryotic cells and their history of safe use in humans. Additionally, RNA, including siRNA, instead of DNA can be delivered using phage virus-like particles (VLPs) [91,92,93]. Hajitou [94] and colleagues developed a chimeric viral vector, AAVP, in which a chimeric genome containing an adeno-associated virus (AAV) transgene cassette is inserted into the phage genome and packaged in an M13 filamentous phage particle that also displayed a eukaryotic cancer cell targeting motif. The RGD-4C targeting peptide facilitates binding of the phage particles to eukaryotic cells by virtue of its binding to αvβ3 integrin proteins which are overexpressed in both tumor cells and the endothelial cells that provide vascular support to many tumors. The inclusion of AAV inverted terminal repeats enhanced transgene expression thus combining a key benefit of eukaryotic viral vectors with the specific cell targeting mediated by the phage. Przystal and colleagues used a related chimeric vector to deliver targeted suicide gene therapy to glioblastoma multiforme intracranial tumors in mice to suppress tumor growth [95]. Like the previous study, the RGD-4C peptide was used to selectively deliver the chimeric viruses to the tumors. Additionally, a tumor specific, drug responsive (temozolomide (TMZ)) promoter (*Grp78*) was used to turn on expression of the suicide transgene (Herpes virus type 1 thymidine kinase (*HSVtk*)) once the cargo was delivered to the tumor cells. The HSVtk enzyme activates the nucleoside analog drug gancylovir, leading to its incorporation during replication which inhibits DNA polymerase activity and leads to cell death by apoptosis. The authors demonstrated a significant synergistic reduction of tumor growth in mice using the chimeric phages combined with both gancylovir and TMZ as compared to either chemotherapy (TMZ) or gene therapy (chimeric phage plus gancylovir) alone. Finally, as CRISPR Cas systems are quickly expanding our ability to edit genetic information, Qazi and colleagues [96] used P22 VLPs to create a programmable delivery vehicle for Cas9 and an sgRNA. By fusing the Cas9 protein to the C terminus of the scaffold protein for P22 they were able to purify self-assembled VLPs from bacteria that had encapsulated both Cas9 and the sgRNA [96]. 

### 4.4. Vaccines

The ability to link genotype with phenotype using phage display technology has made phages a very powerful tool for vaccine and antibody development (for additional reviews see [97,98,99]), but the innate immunogenicity of some phages also makes them useful as vaccine delivery devices [100,101]. The methods described above for gene therapy can readily be applied to DNA [102,103] or RNA based vaccines with the added benefit of the nucleic acid cargo encoding the desired antigen being protected by the phage capsid. Moreover, the ability of some phage capsid proteins to activate the innate immune system obviates the need of an additional adjuvant [64,101,104,105]. When phages displaying protein antigens on their surface are engulfed by antigen presenting cells, they can elicit B-cell and both T-helper and cytotoxic T cell responses to the carried antigens which is especially important when combating viral pathogens [101,106]. T4 bacteriophages have been used to display multiple antigens for HIV, anthrax toxin, foot-and mouth disease virus (FMDV). The results of these preliminary studies demonstrate the ability of phage-based vaccines to elicit both antibody and cell mediated responses (HIV) [107], to neutralize toxins (anthrax) [108], and to protect against infection (FMDV) [109]. Given their innate immunogenicity, some phage-based cancer vaccines may also be able to overcome the immunosuppressive tumor microenvironment. For example, the filamentous phage M13 was engineered to display the extracellular and transmembrane region of a HER2 variant found in the majority of HER2-positive breast cancer patients. Transgenic mice expressing this HER2 variant that were vaccinated with this phage developed fewer tumors, the tumors were smaller, and the latency period was extended likely as a result of overcoming the suppressive tumor microenvironment [110]. Collectively, these results and the results from many additional studies [111,112,113,114,115] indicate that phages might provide a reliable and cost-effective approach for both vaccine development and delivery.

## 5. Genetically Engineered Phages as Sensors

Sensors generally have at least two functional components. First is some recognition element for a target. Second is some transducing mechanism for reporting when the recognition element detects that target. In one sense a bacteriophage is a sensor (or, more exactly, a biosensor). The receptor binding proteins detect the target, a host bacterium. The genome of the phage is the reporter, creating more phages after it enters the target cell, perhaps manifesting as a plaque. In this way, bacteriophages have extraordinary specificity in recognizing their target which can be a strong advantage in a biosensor. However, the reporting component is not necessarily the most practical for a rapidly detecting sensor.

Several approaches use the normal recognition elements of phages to detect bacteria while altering the reporter to make detection more rapid or sensitive. Bacteria are the simplest target as bacteriophages have evolved to bind to them. A number of detection systems simply rely on the reproduction of bacteriophages to indicate the presence of the corresponding host cells [116,117]. These methods are not especially rapid as it generally takes extended culture to detect sufficient bacteriophages either by plaque formation or increases in specific phage proteins. Detection systems that use modified phages have been developed that allow for more rapid reporting. These remain in the research stage but show promise in comparison to traditional pathogen identification using culture methods or genome sequencing.

Some rapid detection systems use phages that have been modified to express a reporter gene such as a bioluminescent protein, e.g., green fluorescent protein (GFP) or luciferase, upon infection [117]. These phages are commonly referred to as reporter phages rather than biosensors, the latter term often reserved for phages affixed to a surface as part of the detector. For both uses though, phages act as sensors with some sort of detectable signal produced following target recognition. Because reporter phage genes are only expressed during infection, detection of the light signal indicates that the phage’s host cell is present. When target bacteria are present in relatively higher concentration, sufficient signal is produced to detect in the first round of infection. For lower concentrations of target bacteria, several rounds of infection can amplify the signal to detectable levels. In some cases, a single colony forming unit (CFU) of bacteria can be detected [118,119].

Another method for detecting bacteria relies not on whole modified phages but rather on phage proteins that have specificity for binding to bacteria. These proteins include the receptor binding proteins (RBPs, components of tail fibers and tail spikes) as well as the cell binding domains (CBDs, also called cell-wall binding domains) of the phage endolysins [67,119]. Each of these binds to specific molecules on the surface of a bacterium with varying levels of specificity. The proteins can be bound or combined as fusion proteins with reporter molecule such as GFP [57,120]. The reporter molecule is then able to specifically bind to a bacterial surface. In another variation, receptor binding proteins or endolysin CBD domains can be bound to surfaces to attach bacteria to those same surfaces. For example, Poshtiban and colleagues [121] used the putative RBP of a *Campylobacter jejuni* phage to functionalize paramagnetic beads that were used to concentrate *C. jejuni* from food samples for detection.

Receptor binding proteins can also be used to detect bacteria by substituting for antibodies in a variation of an ELISA assay designated ELITA (ELISA like tailspike adsorption assay) [122,123]. In these assays, bacteria were adsorbed to microtiter plate wells. The bacteria, either the phage host or controls, were exposed to tailspikes that had been modified to carry a Strep-tag and bound Strep-tagged tailspike was detected with an enzyme linked Strep-tactin probe. In both cases, the goal was to demonstrate tailspike binding, but it should be possible to modify these assays for quantifiable detection of the host bacteria. It would essentially be a sandwich ELISA assay with the tailspike proteins substituting for one of the antibodies.

In addition to being used for sensors for bacteria, as noted in Section 4.1, phages containing novel binding segments derived from phage display can be used to detect specific eukaryotic cells such as tumor cells for imaging and treatment. For example, Askoxylakis and colleagues [124] identified a peptide using phage display that bound to breast cancer cells. After linking the peptide to a fluorescent label, they showed that the peptide targeted cancer cells specifically with the label, suggesting its use for imaging. Linking the peptide to ^125^I created a molecule that preferentially transported the radioisotope to tumor cells in rats carrying the tumor cells. Newton and colleagues [125] developed a targeting peptide via phage display that bound specifically bound the Melanocortin-1 receptor on melanoma cells. They used this to carry a label for melanoma imaging via PET scanning. For additional examples, see reviews by Deutscher [126] and Staquicini [127].

## 6. Genetically Engineered Phages to Facilitate Tissue Construction

Just as phage display can be used to produce phages that bind to specific animal tissues, phage display can be used to create phages containing cell binding peptides that can facilitate tissue construction. There are several potential advantages to the use of phages with cell binding domains or growth promoters as scaffolding components. Phages can self-assemble or undergo templated assembly into 2- and 3-D structures [128,129] or they can be used in 3-D printing to create scaffolds for cells to grow on [130,131]. The most commonly used phages for phage display, filamentous phages, have multiple proteins that can be used for display so that more than one functional peptide can be combined to create multifunctional scaffolds [132]. Finally, phages are easily modified both chemically and genetically ([128]; Section 2 above) and can be cultured at various scales as needed [133].

Phages with these types of cell binding domains have been proposed as functional elements of scaffolds to grow new tissue either in vitro or in vivo [134]. For example, the tripeptide arginine-glycine-aspartic acid (RGD) integrin binding domain has been shown to promote cell adhesion to extracellular matrix fibers and has been studied as an adhesion promoter for a variety of cells [135] Merzlyak and colleagues [136] demonstrated that phage display with an insert library containing an RGD core with varying flanking sequences could be screened to identify phages with improved integrin binding. More significantly, when these phages were allowed to self-assemble into fibers, the fibers promoted the growth of neurons in culture. Chung and colleagues [137] followed up on this work to show that if the same integrin binding motif containing phages were adhered to a surface in an ordered matrix, neurons would also grow along the same matrix. Later work showed that this approach would also guide the growth of fibroblasts [138,139] mesenchymal stem cells (for cartilage production) [140], and osteoblasts [141]. Phage display has also been used to derive many other cell and tissue specific binding peptides (see Table 1 in [134]).

There are many other studies on this use of phages beyond the scope of this brief overview that are can be found in several recent review articles [129,134,142,143].

## 7. Conclusions

Perhaps the best-known therapeutic use of bacteriophages is phage therapy, using phages as antibacterial agents. Typically, phage therapy uses phages as they are found from various environments. But the amenability of phages to modification—either chemically or via genetic engineering—expands the use of phages for much broader applications. Their relatively large genomes (for viruses), structurally complex yet modifiable capsid structure, and rapid replication, all allow for multiple technical applications including the various therapies described in this article and in this special issue. Just as traditional phage therapy remains a standard therapy in some parts of the world and is working toward regulatory approval in other countries, other therapeutic uses of phages will undoubtedly find their way into the clinic in the future. The medical applications for phages and phage-related products are limited only by the imagination of scientists.

## Data Availability

Data sharing not applicable.
